# Identification and Whole Genome Sequencing of the First Case of *Kosakonia radicincitans* Causing a Human Bloodstream Infection

**DOI:** 10.3389/fmicb.2017.00062

**Published:** 2017-01-24

**Authors:** Micah D. Bhatti, Awdhesh Kalia, Pranoti Sahasrabhojane, Jiwoong Kim, David E. Greenberg, Samuel A. Shelburne

**Affiliations:** ^1^Department of Microbiology, MD Anderson Cancer Center, HoustonTX, USA; ^2^Graduate Program in Diagnostic Genetics, School of Health Professions, MD Anderson Cancer Center, HoustonTX, USA; ^3^Department of Infectious Diseases, Infection Control and Employee Health, MD Anderson Cancer Center, HoustonTX, USA; ^4^Quantitative Biomedical Research Center, University of Texas Southwestern Medical Center, DallasTX, USA; ^5^Division of Infectious Diseases, University of Texas Southwestern Medical Center, DallasTX, USA; ^6^Department of Microbiology, University of Texas Southwestern Medical Center, DallasTX, USA; ^7^Department of Genomic Medicine, MD Anderson Cancer Center, HoustonTX, USA

**Keywords:** *Kosakonia radicincitans*, *Enterobacter*, bloodstream infection, AmpC, fimbriae

## Abstract

The taxonomy of *Enterobacter* species is rapidly changing. Herein we report a bloodstream infection isolate originally identified as *Enterobacter cloacae* by Vitek2 methodology that we found to be *Kosakonia radicincitans* using genetic means. Comparative whole genome sequencing of our isolate and other published *Kosakonia* genomes revealed these organisms lack the AmpC β-lactamase present on the chromosome of *Enterobacter* sp. A fimbriae operon primarily found in *Escherichia coli* O157:H7 isolates was present in our organism and other available *K. radicincitans* genomes. This is the first report of a *Kosakonia* species, which are typically associated with plants, causing a human infection.

## Introduction

*Enterobacter* species are major causes of human infections and are particularly prominent nosocomial pathogens due to their broad array of antimicrobial resistance elements (Mezzatesta et al., 2012). Genetic methodologies for analyzing bacterial strains has revealed the complex nature of the *Enterobacter* genus which in turn has resulted in numerous reclassifications of *Enterobacter* and *Enterobacter*-like organisms (Brady et al., 2013). A recent systematic study of strains previously classified as members of the *Enterobacter* genus resulted in the creation of five distinct genera, *Enterobacter, Lelliottia, Pluralibacter, Cronobacter*, and *Kosakonia* (Brady et al., 2013). *E. cloacae* complex strains, which are the most common cause of *Enterobacter* disease in humans, remain in the *Enterobacter* genus (Brady et al., 2013). Clinical microbiology laboratories are increasingly using highly specific techniques such as DNA sequencing and matrix-assisted laser desorption/ionization time-of-flight mass spectrometry (MALDI-TOF) to identify bacterial pathogens (Clark et al., 2013). As reference databases incorporate the recent changes in nomenclature made feasible by such in-depth analyses, there will be an increasing onus on physicians to be familiar with an ever-widening group of organisms. Herein, we report the first infection in a human caused by a *Kosakonia* species and use whole genome analyses of the infecting isolate to identify variant and conserved aspects of virulence and antimicrobial resistance present in *Kosakonia* species relative to *E. cloacae* and other *Enterobacter*-like organisms.

## Case Report

A 61 years old man with cholangiocarcinoma presented to MD Anderson Cancer Center (MDACC) in Houston, TX, USA with a 1 day history of fever, chills, and abdominal pain. Upon admission, he was febrile to 38.5°C, blood pressure was 105/63, heart rate was 111/min, and respiratory rate was 24/min. Physical exam was unrevealing. White blood cell count was 15,000/μl with 89% neutrophils. He was diagnosed with sepsis and admitted to the hospital for therapy with piperacillin-tazobactam. Two sets of blood cultures were both positive for an organism identified as *E. cloacae ssp. cloacae*. Computerized tomography of the abdomen showed no intra-abdominal abscess. He became afebrile on antibiotic therapy and was discharged on a 14 days course of oral ciprofloxacin. One year later, he has had no further infections.

## Materials and Methods

### Ethics Statement

This study was performed using a waiver of informed consent from the MDACC Institutional Review Board, and it was performed in accordance with the approved guidelines.

### Description of MB019

The patient had two blood culture sets obtained at admission that signaled positive for microbial growth at approximately 18 h of incubation. Gram stain performed on the positive culture bottles revealed a gram-negative bacilli, hereafter called MB019, without any unusual characteristics. Overnight subculture of broth from the positive culture bottle yielded gray, smooth colonies on 5% sheep blood agar and very light pink to colorless colonies on MacConkey agar indicating a gram-negative rod with possible weak lactose fermentation. The identification was determined using Vitek2 (BioMériuex), which employs automated biochemical testing. The Vitek2 reported an 87% probability match to *Enterobacter cloacae subsp. cloacae* but noted the isolate was negative for ornithine decarboxylase which is highly unusual for *Enterobacter cloacae* complex members. Antimicrobial susceptibility testing was also performed using the Vitek2 instrument which revealed MB019 to be pan-sensitive including susceptibility to ampicillin, ampicillin/sulbactam, and first and second generation cephalosporins which *Enterobacter cloacae* complex members are considered to be intrinsically resistant per CLSI guidelines. Because of this inconsistent finding, susceptibility testing results were confirmed by a manual method using *E*-test (BioMériuex) which demonstrated that MB019 was pan-susceptible to amikacin, amoxicillin-clavulanate, cefepime, ciprofloxacin, ertapenem, tigecycline, and trimethoprim-sulfamethoxazole at very low minimal inhibitory concentrations. Since the time that MB019 was originally isolated, the MDACC microbiology laboratory has introduced an FDA-cleared, MALDI-TOF MS platform (Vitek MS, BioMériuex) for identifying bacteria. MB019 was recovered from our frozen stocks and the identity and susceptibility profile confirmed by Vitek2. MB019 was also analyzed by the Vitek MS using their FDA approved database and despite five attempts at identification, MALDI-TOF MS was unable to provide an acceptable identification. The closest matches were a split identification between *Cronobacter turicensis* and *Escherichia vulneris*, neither of which had an acceptable identification score. This finding is not surprising since *Kosokonia* spp. are not represented in their current spectral database. Had MB019 been recovered after routine institution of MALDI-TOF, our laboratory protocols would have prompted us to attempt identification using 16S rRNA following the failed identification by MALDI-TOF MS.

#### Whole Genome Characterization

Genomic DNA was isolated from MB019 using the DNeasy Kit (Qiagen) and whole genome sequencing was performed using the Illumina MiSeq (250 bp paired end reads). Two hundred and fifty million base pairs of data were generated. Paired-end reads were aligned to existing genomes using Geneious (version 9.1) with an average sequencing depth of 60x. Genome-wide phylogenetic analysis was performed using the REALPHY pipeline implemented at the MD Anderson Cancer Center High Throughput Computation facility. Briefly, a 120K core- SNP alignment was extracted via mapping of draft or complete genome sequences to reference *K. sacharii* and *E. cloacae* genomes. A maximum-likelihood phylogeny was then reconstructed with PhyML using NNI searches under the GTR+Γ model. Antibiotic resistance genes were identified using RESfinder^1^, and prediction of pathogenicity was performed using the PathogenFinder database^2^. Identification of potential virulence genes was performed via comparative analysis to published *Enterobacter* genomes using Geneious (Liu et al., 2013).

## Results

Blood cultures were both positive for an organism, MB019, originally identified as *E. cloacae ssp. cloacae* via Vitek2 (BioMériuex). MB019 was pan-susceptible to all antimicrobial agents tested and lacked ornithine decarboxylase, which is unusual for *Enterobacter* sp. (Swanson and Collins, 1980). Detailed phenotypic characterization of MB019 is provided in the section “Materials and Methods.” Given the unusual phenotype of MB019, we performed whole genome sequencing via the Illumina MiSeq. Only 479,945 of the generated 737,500 total reads (65%) successfully mapped to the *E. cloacae ssp*. *cloacae* reference strain ATCC 13047 indicating that the organism was unlikely to actually be a member of the *E. cloacae* complex. The closest match by 16s rRNA sequencing using BLASTn was *Kosakonia radicincitans* with 1524/1529 sequence identity (99%) whereas there was 1514/1543 (98%) sequence identity with the 16s rRNA of ATCC 13047 (Ren et al., 2010). Similarly, the Silva database^3^ identified the 16s rRNA of MB019 as being derived from *Kosakonia*. Using the short-read data, we identified a single putative 35 kb plasmid with limited homology to plasmids present in other gram-negative bacteria such as plasmid BK0683 from *Klebsiella pneumoniae*. No antimicrobial resistance elements were identified on this putative plasmid.

**FIGURE 1:**
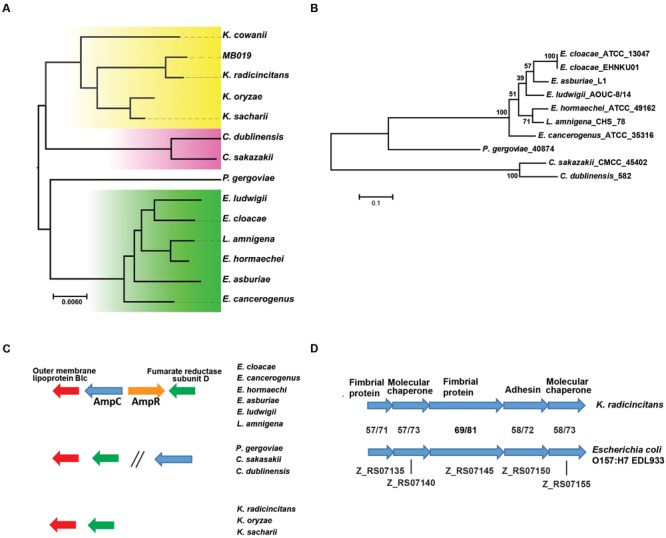
**Whole genome characterization of MB019 and other *Kosakonia* strains. (A)** Whole genome phylogenetic analysis was performed in PhyML as described in the manuscript. The following strains with NCBI accession numbers in parentheses are included: *E. cloacae* ATCC 13047 (NC_014121.1); *E. asburiae* LF7a (NC_015968.1); *E. cancerogenus* ATCC 35316 (GCA_000155995.1); *E. hormaechei* ATCC 49162 (GCA_000213995.1); *E. ludwigii* EN-119 (GCA_000818595.1); *L. amnigena* CHS 78 (GCA_000694955.1); *P. gergoviae* FB2 (NZ_CP009450.1). *C. sakazakii* ATCC BAA-894 (NC_009778.1); *C. dubliniensis* LMB 23823 (NZ_CP01266.1); *K. radicincitans* DSM 16656 (GCA_000280495.1); *K. cowanii* JCM_10956 (GCA_001312885.1); *K. sacharii* (NZ_CP007215.1); *K. oryzae* (GCA_000958895.1). **(B)** Phylogenetic analysis of AmpC homologs present in *Enterobacter* and *Enterobacter*-like organisms. AmpC homologs were identified using BLASTp, aligned via MUSCLE, and a phylogenetic tree was generated using MEGA v. 6 using nearest neighbor setting. Bootstrapping was performed using 1000 iterations with numbers reflecting confidence of branching. Inset shows genetic distance. **(C)** Analysis of genetic region of AmpC encoding genes in *Enterobacter* and *Enterobacter*-like organisms. Genes are color coded with genetic arrangements grouped by indicated organisms. **(D)** Identification of fimbriae operon present in *K. radicincitans* and *E. coli* O157:H7 strains. Putative functions of encoded genes are shown. Numbers indicate percent identity and similarity, respectively, at the amino acid level for each protein between MB019 and *E. coli* 0157:H7 EDL933 (NC_02655).

Currently there are ten *Kosakonia* genomes in the NCBI database, three of *K. radicincitans*, two each of *K. sacchari* and *K. oryzae*, and one each of *K. cowanii, K. oryziphila*, and *K. oryzendophytica*, although the *K. sacchari* strain has not been clearly assigned to the *Kosakonia* genus (Brady et al., 2013). This analysis clearly identified strain MB019 as being most closely related to *K. radicincitans* (**Figure 1A**). In general our whole genome phylogeny was in accordance with the recent multi-locus sequence analysis (MLSA) proposed by Brady et al. (2013) although *Lelliottia amnigena*, an MLSA group B strain, was admixed with *Enterobacter* strains of MLSA group A (**Figure 1A**).

The ability of *Enterobacter* strains to cause nosocomial infections is thought to be in large part due to their intrinsic antimicrobial resistant properties, such as the chromosomally encoded AmpC β-lactamase (Mezzatesta et al., 2012). Consistent with the phenotypic analysis, no genes encoding known antimicrobial resistance elements were detected in MB019 via RESfinder. Thus, we sought to determine the presence of AmpC in strains that until recently were classified in the genus *Enterobacter*. Interestingly, strains of all of the MLSA groups contained AmpC homologs except for the *Kosakonia* genus (**Figure 1B**). The MLSA group A and B strains contain the same gene arrangement with *ampC* and *ampR*, which encodes an *ampC* regulator, flanked 5′ and 3′ by genes encoding the outer membrane protein Blc and fumarate reductase subunit D, respectively (**Figure 1C**). For *Pluralibacter gergoviae* (MLSA group C) and *Cronobacter sakazakii* (MLSA group E), genes encoding Blc and fumarate reductase subunit D are immediately adjacent, *ampC* is located at a distant site, and *ampR* is absent (**Figure 1C**). Similar to *Pluralibacter* and *Cronobacter*, Blc and fumarate reductase subunit D encoding genes are immediately adjacent in *Kosakonia* genus strains but *ampC* and *ampR* are not present (**Figure 1C**).

Given that *Kosakonia* strains have not previously been reported to cause human disease, we submitted the MB019 genome to the PathogenFinder database^4^, which predicted MB019 to be a human pathogen with a confidence of 0.65. We next searched for possible virulence determinants present in the MB019 genome. MB019 contains homologs of one of the two type IV secretion system present in *E. cloacae subsp. cloacae* ATCC13047 (Liu et al., 2013) (**Table 1**). Similarly, MB019 contains only homologs of one of the two type VI secretion systems present in ATCC13047 (**Table 1**). Fimbriae proteins are considered important mediators of adhesion in *Enterobacteriaceae* and there are 13 fimbriae operons present in *E. cloacae* ATCC13047, none of which are present in MB019 or other *Kosakonia* genomes (Wurpel et al., 2013). MB019 contains two putative fimbriae encoding operons (**Table 1**), one of which has significant homology (amino acid identity 61%, similarity 75% over the length of the operon) with an identically arranged fimbriae operon present in *Escherichia coli* O157:H7 strains (**Figure 1D**). Interestingly, this operon, whose function is unknown and has been variously labeled as *loc6* or “type 3-like,” appears to be limited to *E. coli* phylogroup E strains, which includes O157 and O26 strains (Low et al., 2006; Wurpel et al., 2013). Although the operon is present and highly conserved amongst all three *K. radicincitans* strains present in NCBI and in strain MB019, the operon is not present in other sequenced *Kosakonia* species.

**Table 1:** Potential virulence factor encoding genes present in MB019.

Putative function of encoded protein(s)	Genome position^a^	Presence/Function of paralogs in other bacteria
Fimbriae operon	Y71_RS05635-05645	Not identified
	Y71_RS18325-18345	*E. coli* O157:H7 EDL933 *loc6* (Z_RS07135-07155), unknown function
Type IV secretion system	Y71_RS16795-16845	*E. cloacae* ATCC_13047 (ECL00414-ECL00424), pilus biogenesis
Type VI secretion system	Y71_RS12200-12295	*E. cloacae* ATCC_13047 (ECL01532-01552), protein delivery system
Enterobactin synthesis	Y71_RS22265-22330	*E. cloacae* ATCC_13047 (ECL03104-03118), siderophore production

*^a^Refers to genome position in *K. radicincitans* DSM 16656.*

## Discussion

Herein, we report the first case of a human infection caused by a *Kosakonia* genus organism, specifically *K. radicincitans*. Previously known as *Enterobacter radicincitans*, all prior reports of this organism have described it as a plant associated bacteria and have mainly focused on its growth promoting properties (Kampfer et al., 2005; Schreiner et al., 2009; Witzel et al., 2012; Brock et al., 2013; Suhaimi et al., 2014; Bergottini et al., 2015). However, there is a report of *K. radicincitans* causing bacterial wilt disease in bananas, suggesting its pathogenic potential (Suhaimi et al., 2014). Given the difficulties in accurately identifying organisms previously labeled as part of the *Enterobacter* genus, we cannot determine whether this is actually the first case of human infection due to *K. radicincitans* or whether the organism has been previously misidentified, as originally occurred with our isolate.

A key finding of our whole genome analysis was that all *Kosakonia* genus strains sequenced to date lack the *ampC* gene, which is chromosomally encoded on *Enterobacter* genus strains and can confer resistance to a broad array of β-lactam agents when de-repressed (Mezzatesta et al., 2012). Given the presence of AmpC in *Enterobacter* strains, it is often recommended to treat such infections with carbapenem type antibiotics given the stability of these compounds even in the setting of high AmpC production (Mezzatesta et al., 2012). Inasmuch as *Kosakonia* strains lack AmpC, and other known antimicrobial resistance mechanisms, accurate identification of these organisms is needed to ensure clinicians utilize more targeted antimicrobials.

A surprising result of our search for virulence determinants of *Kosakonia* species was the finding of a fimbriae operon with significant homology to one present in *E. coli* O157:H7 strains. This operon was not present in other *Enterobacter* or *Enterobacter*-like organisms suggesting that lateral gene transfer has occurred between *E. coli* and *Kosakonia* species. The transfer of fimbriae synthesis proteins has been observed between intestinal Bacteroidales strains as such exchange is thought to facilitate ecological advantages (Coyne et al., 2014). How an organism typically associated with plants would come to share genetic material with a gut commensal is not clear, but we hypothesize that the presence of this operon may have facilitated the initial colonization of our patient by strain MB019.

In summary, we have used whole genome sequencing to identify and characterize the first human infection due to *Kosakonia radicincitans*. Further study of strains being identified as *Enterobacter* by the clinical microbiology laboratory may reveal additional infections due to this newly described pathogen.

## Author Contributions

MB and PS carried out the microbiologic investigations. AK, JK, DG, and SS performed the genomic analyses. MB, AK, and SS wrote the paper. All authors read and approved the final manuscript.

## Conflict of Interest Statement

The authors declare that the research was conducted in the absence of any commercial or financial relationships that could be construed as a potential conflict of interest.

## Footnotes

1 https://cge.cbs.dtu.dk/services/ResFinder

2 https://cge.cbs.dtu.dk/services/PathogenFinder

3 http://www.arb-silva.de/

4 https://cge.cbs.dtu.dk/services/PathogenFinder
